# Membrane-Fluidization-Dependent and -Independent Pathways Are Involved in Heat-Stress-Inducible Gene Expression in the Marine Red Alga *Neopyropia yezoensis*

**DOI:** 10.3390/cells11091486

**Published:** 2022-04-28

**Authors:** Ho Viet Khoa, Koji Mikami

**Affiliations:** 1 Graduate School of Fisheries Sciences, Hokkaido University, 3-1-1 Minato-cho, Hakodate 041-8611, Japan; hvkhoa59@gmail.com; 2 Department of Integrative Studies of Plant and Animal Production, School of Food Industrial Sciences, Miyagi University, 2-2-1 Hatatate, Sendai 982-0215, Japan

**Keywords:** heat stress, gene expression, membrane fluidity, Bangiales cysteine-rich motif, *Neopyropia yezoensis*

## Abstract

Heat stress responses are complex regulatory processes, including sensing, signal transduction, and gene expression. However, the exact mechanisms of these processes in seaweeds are not well known. We explored the relationship between membrane physical states and gene expression in the red alga *Neopyropia yezoensis*. To analyze heat-stress-induced gene expression, we identified two homologs of the heat-inducible *high temperature response 2* (*HTR2*) gene in *Neopyropia seriata*, named *NyHTR2* and *NyHTR2L*. We found conservation of *HTR2* homologs only within the order Bangiales; their products contained a novel conserved cysteine repeat which we designated the Bangiales cysteine-rich motif. A quantitative mRNA analysis showed that expression of *NyHTR2* and *NyHTR2L* was induced by heat stress. However, the membrane fluidizer benzyl alcohol (BA) did not induce expression of these genes, indicating that the effect of heat was not due to membrane fluidization. In contrast, expression of genes encoding multiprotein-bridging factor 1 (*NyMBF1*) and HSP70s (*NyHSP70-1* and *NyHSP70-2*) was induced by heat stress and by BA, indicating that it involved a membrane-fluidization-dependent pathway. In addition, dark treatment under heat stress promoted expression of *NyHTR2*, *NyHTR2L*, *NyMBF1*, and *NyHSP70-2*, but not *NyHSP70-1*; expression of *NyHTR2* and *NyHTR2L* was membrane-fluidization-independent, and that of other genes was membrane-fluidization-dependent. These findings indicate that the heat stress response in *N. yezoensis* involves membrane-fluidization-dependent and -independent pathways.

## 1. Introduction

Increases in seawater temperature reduce the productivity and quality of economically important red algae of the order Bangiales, such as *Neopyropia yezoensis* and *Neopyropia haitanensis* (formerly known as *Pyropia yezoensis* and *Pyropia haitanensis*, respectively) [1,2]. Recent comparative transcriptome analyses have demonstrated that these algae respond to heat stress through alteration of gene expression profiles [3,4], indicating that red algae have heat-inducible genes. Indeed, heat-inducible expression of genes encoding heat shock protein 70 (HSP70) and nuclear localizing high temperature response 2 (HTR2) have been confirmed in *N. yezoensis*, *N. haitanensis*, and *Neopyropia seriata* (formerly *Pyropia seriata*) [5,6,7,8]. In addition, the presence of an intrinsic ability to memorize non-lethal heat stress, which enables survival under lethal temperature by establishment of heat stress tolerance, was confirmed in ‘*Bangia*’ sp. ESS1 [9]; however, other ‘*Bangia*’ species lack this heat stress memory [10]. Therefore, elucidation of mechanisms regulating responses to heat stress and acquisition of heat stress tolerance based on heat stress memory is indispensable for building strategies to protect the economically important Bangiales from seawater warming.

Responsibility to heat stress with changes in the gene expression pattern, morphology, and composition of metabolites is evolutionally conserved in green lineage including terrestrial plants and their closest algal relatives [11,12,13,14,15]. However, it seems that there are both conserved and diversified parts in the regulatory mechanisms of these physiological and molecular responses to heat stress among Archaeplastida. Indeed, in terrestrial plants, heat-inducible transcription of the gene encoding of the class A3 heat shock factor (HsfA3), which is a transcription factor regulating mRNA expression of heat shock protein (*HSP*) genes, is controlled by the APETALA2 (AP2) domain transcription factor dehydration-responsive element-binding protein 2A (DREB2A) [16,17,18]. In addition, expression of DREB2A is heat-inducible through regulation by the class A1 Hsfs, such as HsfA1a, HsfA1b, HsfA1d, and HsfA1e [18,19]. These findings indicate that the heat-stress-inducible expression of HSP genes involves a stepwise activation cascade of transcription factors. However, genome and extensive transcriptome analyses have clearly demonstrated a lack of AP2-domain-containing proteins in red algae, suggesting that the heat signal transduction pathways in Bangiales differ from those in terrestrial plants. Moreover, although the presence of the *HSF* gene was confirmed in Bangiales, including *N. yezoensis* and *Porphyra umbilicalis* [20], as well as in unicellular red algae and Florideophyceae [20,21], little is known about the activation mode and function of *HSF* in red algae. Regulatory mechanisms of heat stress responses in Bangiales remain unknown.

Heat stress increases plant membrane fluidity [22,23]. Therefore, homeostasis of the membrane physical state under heat stress is balanced by an increase in saturation level due to accumulation of saturated fatty acids, which increases membrane rigidity and lowers membrane fluidity [24,25,26,27,28]. Thus, sensing of membrane fluidization is thought to trigger plant heat stress responses [29,30,31,32,33]. Evidence for this includes an artificial increase in the saturation level of membrane fatty acids following pharmacological treatment with the membrane fluidizer benzyl alcohol (BA) [34,35,36] and the genetic inactivation of fatty acid desaturase and glycerol-3-phosphate acyltransferase genes in terrestrial plants [37,38,39]. In ‘*Bangia*’ sp. ESS1 (order: Bangiales), a moderate increase in the saturation rate of membrane fatty acids under heat stress conditions promotes acquisition of heat stress tolerance through the establishment of heat stress memory [9]. However, it is unknown whether heat-stress-induced gene expression is initiated by changes in membrane fluidity.

In the present study, we analyzed the relationship between membrane fluidization and the expression of genes encoding novel cysteine-rich domain proteins (homologs of HTR2 of *N. seriata*; [6]), HSP70s [5], and multiprotein-bridging factor 1 (MBF1; [40]) in the red alga *N. yezoensis*. Our findings demonstrate that both membrane-fluidization-dependent and -independent pathways regulate heat-inducible gene expression and that these pathways can be enhanced by dark conditions. These findings provide insights into the triggers of heat stress responses in poikilothermic organisms.

## 2. Materials and Methods

### 2.1. Algal Materials and Culture Conditions

Gametophytes, sporophytes, and conchosporophytes of the *N. yezoensis* (strain U-51) were maintained in sterilized artificial seawater (SEALIFE, Marinetech, Tokyo, Japan) enriched with ESS2 [41] under 60–70 μmol photons m^−2^ s^−1^ light with a short-day photoperiod (10 h light/14 h dark) at 15 °C with air filtered through a 0.22 μm filter (Whatman, Maidstone, UK). The culture medium was changed weekly.

Three life cycle generations were subjected to temperature stress, which commenced at 12:00, 3 h after the start of light irradiation (9:00). Stress treatment was performed by incubating algae at 5, 15, and 25 °C for 0.5, 1, 2, 4, 6, 8, and 12 h. Since the duration of light irradiation was only 10 h, the 8 and 12 h incubations included 1 and 5 h of dark conditions, respectively. Experiments were also carried out with 24 h light.

Pharmacological treatment with benzyl alcohol (BA) and dimethyl sulfoxide (DMSO) was performed by incubating algae at 15 °C for 5, 15, and 30 min. Incubations at 25 °C for 8 and 12 h under light with BA or dark conditions with DMSO were also performed. Temperature-stressed and pharmacologically treated gametophytes, sporophytes, and conchosporophytes were harvested, frozen in liquid nitrogen, and stored at −80 °C prior to use for gene expression analysis.

### 2.2. Identification and Phylogenetic Analysis of HTR2 Homologs

**Figure 1:**
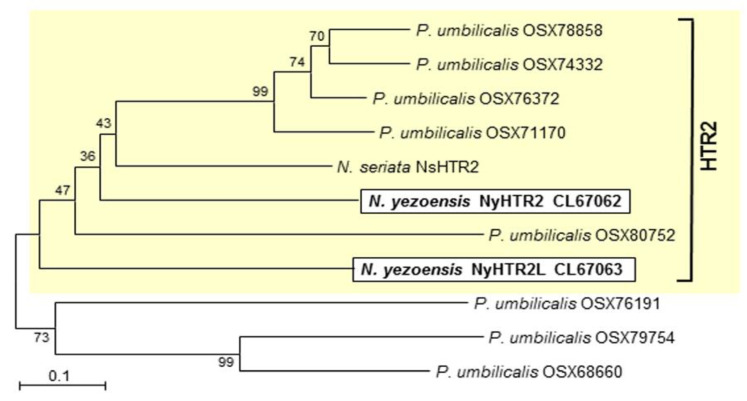
HTR2 homologs are conserved in the Bangiales. DDBJ/EMBL/GenBank accession numbers of homologs are indicated after species names. HTR2 homologs from *Neopyropia yezoensis* are highlighted and boxed. The bootstrap values with 1000 replicates are indicated at the nodes of the tree. Bar, 0.1 substitutions per site.

A homology search using our *N. yezoensis* transcriptome data [42] was performed against the *NsHTR2* amino acid sequence to find candidate *NsHTR2* homologs. The ORF finder (https://www.ncbi.nlm.nih.gov/orffinder/ (access on 30 March 2020)) was used to identify full-length open reading frames (ORFs) of these candidates. A BLAST search (https://blast.ncbi.nlm.nih.gov/Blast.cgi (access on 30 march 2020)) using predicted amino acid sequences of *NsHTR2* and candidates from *N. yezoensis* as queries was performed to find HTR2 homologs from other algae and terrestrial plants. The amino acid sequences of all the obtained HTR2 homologs were used to construct a neighbor-joining phylogenetic tree with MEGA 7 (https://www.megasoftware.net (access on 1 December 2021)) using ClustalW to align the sequences. The accession numbers of the sequences, except for *NsHTR2*, from DDBJ/EMBL/GenBank are shown next to the species names in the phylogenetic tree in Figure 1.

### 2.3. Total RNA Extraction, DNA Removal, and cDNA Synthesis

Extraction of total RNA from gametophytes, sporophytes, and conchosporophytes, removal of genomic DNA contamination, quality check of purified total RNA, and synthesis of first-strand complementary DNA (cDNA) were performed as described in [41]. The thermal cycling parameters for the first-strand cDNA synthesis consisted of an initial denaturation step at 98 °C for 30 s; 30 cycles of 98 °C for 10 s, 60 °C for 30 s, and 72 °C for 20 s; and a final extension step at 72 °C for 5 min.

### 2.4. Quantitative Gene Expression Analysis

Primers for qPCR were designed using Primer Premier 5 (http://www.premierbiosoft.com (access on 2 April 2020)) and confirmed productivity of PCR products with expected sizes according to [41]. We used two real-time PCR machines. The thermal cycling parameters for the Applied Biosystems 7300 real-time PCR system (Life Technologies, Carlsbad, CA, USA) consisted of 95 °C for 5 min and 40 cycles of 94 °C for 30 s, 60 °C for 30 s, and 72 °C for 20 s. The thermal cycling parameters for the AriaMX (3000P) real-time PCR system (Agilent Technologies, Santa Clara, CA, USA) consisted of 95 °C for 3 min and 40 cycles of 95 °C for 5 s and 60 °C for 20 s. A dissociation curve was generated to check for specificity of amplification by heating from 60 to 95 °C in the Applied Biosystems 7300 real-time PCR system or by 95 °C for 1 min, 55 °C for 30 s, and 95 °C for 30 s in the AriaMX (3000P) real-time PCR system.

### 2.5. Statistical Analysis

Values are presented as the mean ± SD from triplicate experiments. Two-way ANOVA followed by a Tukey–Kramer test was used for multiple comparisons, and significant differences were determined using a cutoff value of *p* < 0.05.

## 3. Results

### 3.1. Discovery of a Novel Protein Family Specifically Conserved in Bangiales

By homology searching of the transcriptome data of *N. yezoensis* [42], we found two homologs (Unigene16775 and CL1219.Contig1) of *NsHTR2*, which has been identified as a heat-inducible gene in *N. seriata* [6]. In addition, we identified eight homologs (OSX78858, OSX74332, OSX76372, OSX71170, OSX80752, OSZ76191, OSX79754, and OSX68660) from the red alga *Porphyra umbilicalis* [43] through an NCBI BLAST search with *NsHTR2* as a query. We found no other homologs among florideophyceans, green and brown algae, or terrestrial plants, indicating that HTR2 is a Bangiales-specific protein. Phylogenetic analysis using amino acid sequences of the proteins revealed two clades (Figure 1): an HTR2 clade containing all *Neopyropia* proteins and five *P. umbilicalis* proteins (OSX78858, OSX74332, OSX76372, OSX71170, and OSX80752) and a clade of HTR2-related proteins that contains OSZ76191, OSX79754, and OSX68660 from *P. umbilicalis*. Since Unigene16775 is more closely related to *NsHTR2* than to CL1219.Contig1, we designated Unigene16775 and CL1219.Contig1 as *NyHTR2* and *NyHTR2L* (HTR2-like), respectively. The DDBJ/EMBL/GenBank accession numbers of *NyHTR2* and *NyHTR2L* are LC672062 and LC672063, respectively. It is unknown whether *N. seriata* has other HTR2 homologs.

Amino acid alignment indicated that all the HTR2 homologs have a unique domain at their C-terminal half in which the positions of 10 cysteine (Cys) residues were highly conserved (Figure S1). Cross-brace zinc finger motifs, such as RING, PHD, FYVE, and ZZ fingers, are structural features that bind zinc atoms through eight Cys and/or His ligands, each with a specific biological role [44,45]. However, the Cys-rich domains of the HTR2 proteins differed from previously identified cross-brace zinc fingers and did not show sequence similarity to any conserved domains based on three-dimensional structure predictions with the Phyre2 Server (http://www.sbg.bio.ic.ac.uk/phyre2/html/ (access on 1 December 2021)). Thus, HTR2 homologs are novel proteins containing an unknown Cys-repeat motif. Since it is uncertain whether this motif is a new type of cross-brace zinc finger, we designated it the Bangiales cysteine-rich (BCR) motif.

### 3.2. Determination of a Reference Gene for Quantitative Analysis of Heat-Inducible Gene Expression in Different Life Cycle Generations of N. yezoensis

We selected three candidate reference genes for qRT-PCR analysis of heat-inducible gene expression in three generations of the *N. yezoensis* life cycle, i.e., gametophyte, sporophyte, and conchosporophyte: *GAPDH* (glyceraldehyde 3-phosphate dehydrogenase), *EF1α* (Elongation factor1-α), and *eIF4A* (Translation initiation factor 4A). These were previously proposed as reference genes for qRT-PCR in *N. yezoensis* [46,47]. We validated the specificity and efficiency of the newly designed primer pairs (Table S1) by 2% agarose gel electrophoresis of amplicons of each candidate gene derived from cDNA libraries of gametophytes, sporophytes, and conchosporophytes. A single PCR fragment with expected size was amplified, which gave rise to a single peak in the melting curve (data not shown). The amplification efficiency (E) of each set ranged from 95.13 to 102.55%, and the linear correlation coefficients (R^2^) ranged from 0.9948 to 0.9973 (Table S1), both of which were within the acceptable range (E: 90–105%; R^2^: 0.9910–0.9998). Moreover, we were able to generate standard curves for all primer sets using a tenfold dilution series with six dilution points in triplication (data not shown). Thus, all primer sets are suitable for further characterization of candidates as desired reference genes.

We calculated the expression stability of these candidates by measuring the cycle threshold (Ct) values of the genes. We synthesized template cDNAs for qRT-PCR using total RNAs separately prepared from three generations, each of which were a mixture of one control sample and triplicate samples treated with 5, 15, or 25 °C for 0.5, 1, 2, 4, 6, 8, or 12 h for each generation (a total of 22 samples for each generation). The Ct values of reference genes ranged from 17.4 to 23.8, 16.7 to 23.2, and 18.3 to 24.3 in gametophytes, sporophytes, and conchosporophytes, respectively (Figure S2A). Of the candidates, *EF1α* displayed the lowest expression level, while both *GAPDH* and *eIF4A* had the highest expression levels (Figure S2A). Next, we used geNorm software to rank the expression stability of each gene by calculating the expression stability value. Figure S2B shows the average expression stability (M) of three candidates. *eIF4A* was the most stable, with M < 1.5 in all of generations. Moreover, *eIF4A* was ranked as the most stable gene in all three life cycle generations using NormFinder (Table S2). Therefore, we concluded that *eIF4A* is a suitable reference gene for normalizing gene expression under both heat and cold stress conditions in all life cycle generations.

To confirm the above findings, we subjected all the candidate reference genes to qRT-PCR to examine *NyMBF1* expression across *N. yezoensis* generations under heat (25 °C) and cold (5 °C) stress (see Table S3 for primers for *NyMBF1*). Our rationale was that it seemed plausible that *NyMBF1* expression might be induced by heat stress, as occurs with numerous genes in terrestrial plants [48]. We incubated algae from each of the three generations separately at 5, 15, or 25 °C for 0.5, 1, 2, 4, 6, 8, or 12 h. When we normalized qRT-PCR data using *eIF4A* as the reference gene, it was clear that heat (but not cold) induced the expression of *NyMBF1* in all three generations, with peaks at 2 and 8 h after exposure to 25 °C. In contrast, when we normalized the data using *GAPDH* or *EF1α*, we did not observe heat-dependent expression of *NyMBF1* (Figures S3–S5, Table S4). These findings indicate that *NyMBF1* is a heat-stress-inducible gene, and *eIF4A* is a suitable reference gene to use in monitoring gene expression under heat and cold stress in the three generations of *N. yezoensis*. Accordingly, we employed *eIF4A* as a reference gene in the rest of our study.

### 3.3. Heat-Inducible and Dark-Stimulated Expression of NyHTR2 and NyHTR2L

**Figure 2:**
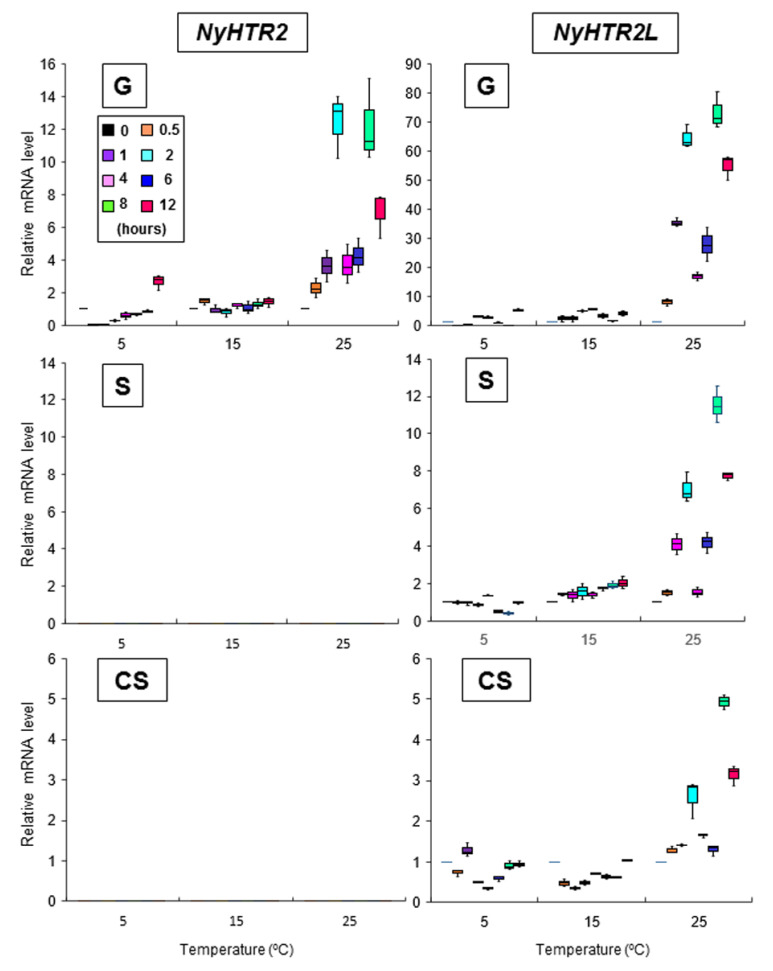
Temperature-dependent expression patterns of *NyHTR2* and *NyHTR2L* with temporal comparisons over three life cycle generations of *Neopyropia yezoensis*. The relative mRNA levels were normalized using *eIF4A* as the reference gene. In the box plots, values on the Y axis represent the fold change of relative quantification of each gene. Since no expression of *NyHTR2* was observed in sporophyte and conchosporophyte, nothing is displayed in corresponding panels in the figure. Significant differences in expression level among the three life cycle generations from triplicate independent replicates as defined by Tukey’s test (*p* < 0.05) in two-way ANOVA are shown in Table S5. G—gametophyte; S—sporophyte; CS—conchosporophyte.

Since HTR2 was identified as a heat-inducible gene in *N. seriata* [6], we analyzed expression profiles of both *NyHTR2* and *NyHTR2L* under the same cold and heat stress conditions as those used to analyze *NyMBF1* (Figures S3–S5 and Table S4) using primers, as shown in Table S3. *NyHTR2* was expressed only in gametophytes and was induced by heat stress (Figure 2, Table S5). *NyHTR2* expression peaked at 2 and 8 h after exposure to 25 °C (Figure 2, Table S5). Since *N. yezoensis* was cultured under a short-day photoperiod (10 h light: 14 h dark) and the temperature treatments began 3 h into the light period, the *NyHTR2* expression peak at 8 h occurred 1 h after the start of the dark period. Therefore, the first and second *NyHTR2* expression peaks occurred under heat and heat–dark conditions, respectively. Moreover, *NyHTR2L* was induced by heat stress in all three generations, with expression peaks at 2 and 8 h after exposure to 25 °C heat stress (Figure 2). *NyMBF1* expression followed the same pattern in all three generations (Figures S3–S5, Table S4). Therefore, the heat-inducible expression patterns of *NyHTR2*, *NyHTR2L*, and *NyMBF1* were identical; although, temporal expression patterns of *NyHTR2* during the life cycle differed from those of other genes.

We also reexamined the heat-inducible expression of *NyHSP70-1* and *NyHSP70-2* (see Table S3 for primers), which had been demonstrated previously [5]. In gametophytes, both genes had expression peaks at 2 h after heat exposure. *NyHSP70-2*, but not *NyHSP70-1*, also showed a second peak at 8 h after exposure (Figure S6, Table S6). We observed similar expression patterns in sporophytes and conchosporophytes. Expression peaks in conchosporophytes occurred rapidly, at 1 h after exposure (Figure S6, Table S6). These findings indicate that the appearance of a second expression peak under heat–dark conditions varies depending on the gene.

**Figure 3:**
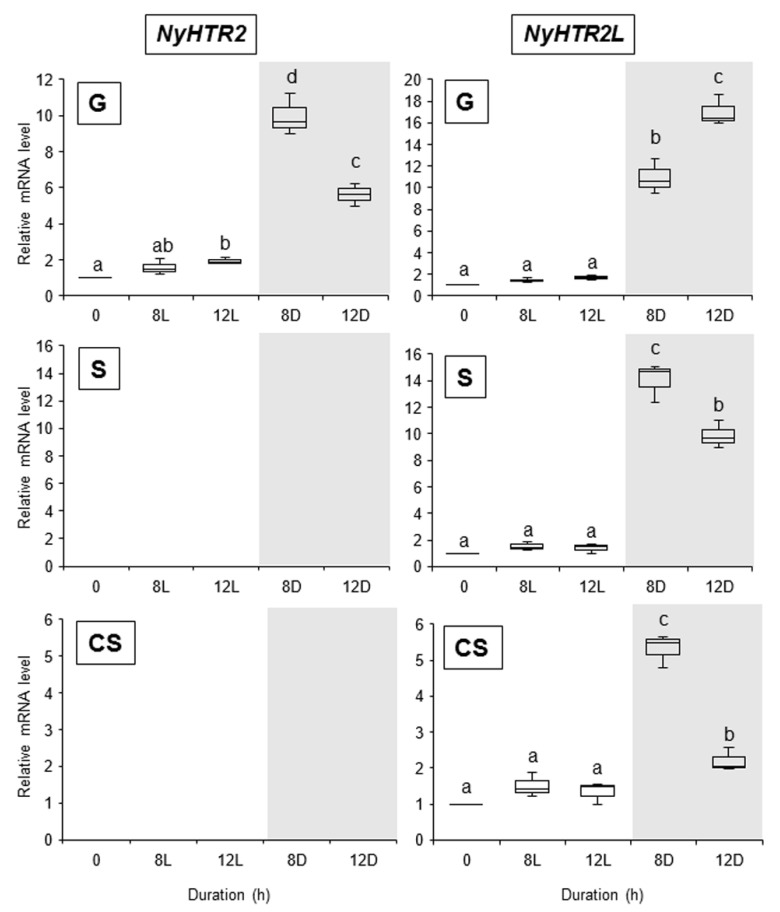
Effect of dark treatment on the second expression peak of *NyHTR2* and *NyHTR2L* in three life cycle generations of *Neopyropia yezoensis* at 25 °C. Gene expression in the light and the dark, at 8 and 12 h after heat stress exposure, was compared. Relative mRNA levels were normalized using *eIF4A* as the reference gene. In the box plots, values on the Y axis represent the fold change of relative quantification of each gene. Since no expression of *NyHTR2* was observed in sporophyte and conchosporophyte, nothing is displayed in corresponding panels in the figure. Different alphabetical letters denote significant differences in expression level among the three life cycle generations from triplicate independent replicates as defined by Tukey’s test (*p* < 0.05) in two-way ANOVA. L—light; D—dark; G—gametophyte; S—sporophyte; CS—conchosporophyte.

**Figure 4:**
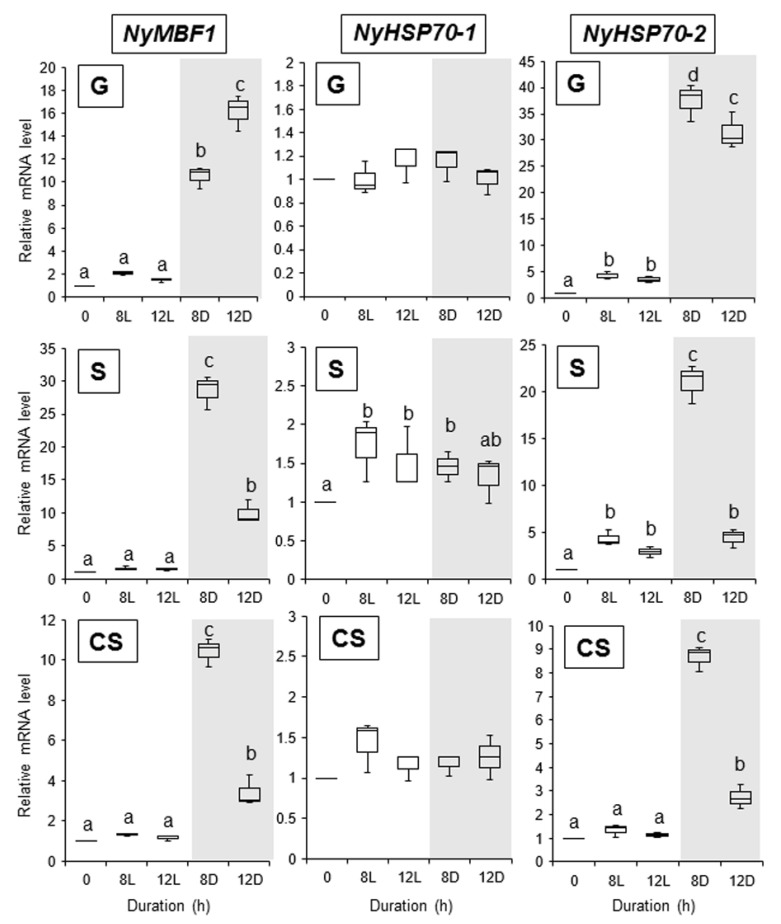
Effect of dark treatment on the second expression peak of *NyMBF1*, *NyHSP70-1*, and *NyHSP70-2* in three life cycle generations of *Neopyropia yezoensis* at 25 °C. Gene expression in the light and dark, and at 8 and 12 h after heat stress exposure, were compared. Relative mRNA levels were normalized using *eIF4A* as the reference gene. In the box plots, values on the Y axis represent the fold change of relative quantification of each gene. Different alphabetical letters denote significant differences in expression level among the three life cycle generations from triplicate independent replicates as defined by Tukey’s test (*p* < 0.05) in two-way ANOVA. L—light; D—dark; G—gametophyte; S—sporophyte; CS—conchosporophyte.

To further explore the drivers of the second expression peak, we changed the culture conditions to 24 h light. Under these conditions, we did not observe a second expression peak for *NyHTR2*, *NyHTR2L*, *NyMBF1*, or *NyHSP70-2* in any generation (8 L and 12 L in Figure 3 and Figure 4). There was no expression peak for *NyHSP70-1* under either regular or 24 h light conditions (Figure 3 and Figure 4). These findings indicate that the second expression peak requires darkness as well as heat stress. Thus, dark conditions result in a second, heat-stress-dependent induction of *NyHTR2*, *NyHTR2L*, *NyMBF1*, and *NyHSP70-2* expression after the first transient expression, which is inhibited by light illumination.

### 3.4. Relationship between Heat-Inducible Expression and Membrane Fluidization

Membrane fluidization, caused by heat stress, triggers heat-induced gene expression in terrestrial plants [29,30,31,32,33]; however, it is unknown whether changes in the membrane’s physical state are related to gene expression in seaweeds. Thus, we examined the effects of a membrane fluidizer, benzyl alcohol (BA), and a membrane rigidizer, dimethyl sulfoxide (DMSO), on the expression of heat-inducible genes.

**Figure 5:**
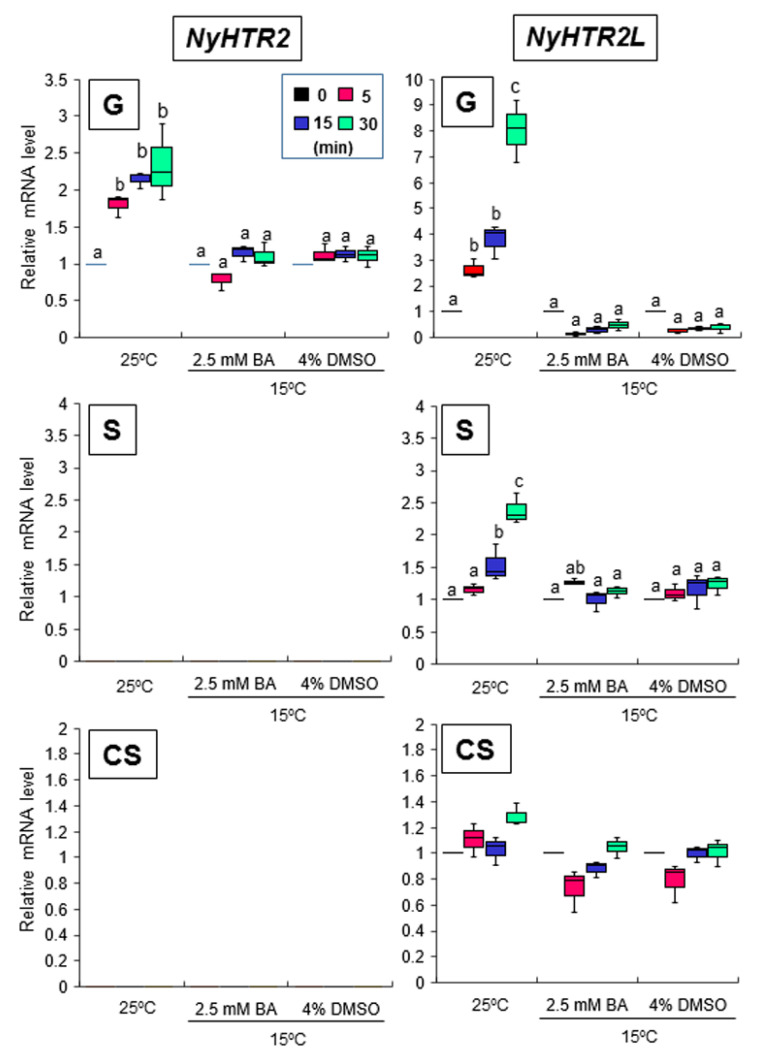
Effects of changes in membrane physical state on *NyHTR2* and *NyHTR2L* expression in three life cycle generations of *Neopyropia yezoensis*. The algae were treated with 2.5 mM BA and 4% DMSO at 15 °C for 5, 15, and 30 min, and the expression of *NyHSP70-1* and *NyHSP70-2* was compared with their expression at 25 °C. Relative mRNA levels were normalized using *eIF4A* as the reference gene. In the box plots, values on the Y axis represent the fold change of relative quantification of each gene. Since no expression of *NyHTR2* was observed in sporophyte and conchosporophyte, nothing is displayed in corresponding panels in the figure. Different alphabetical letters denote significant differences in expression level among the three life cycle generations from triplicate independent replicates as defined by Tukey’s test (*p* < 0.05) in two-way ANOVA. G—gametophyte; S—sporophyte; CS—conchosporophyte.

**Figure 6:**
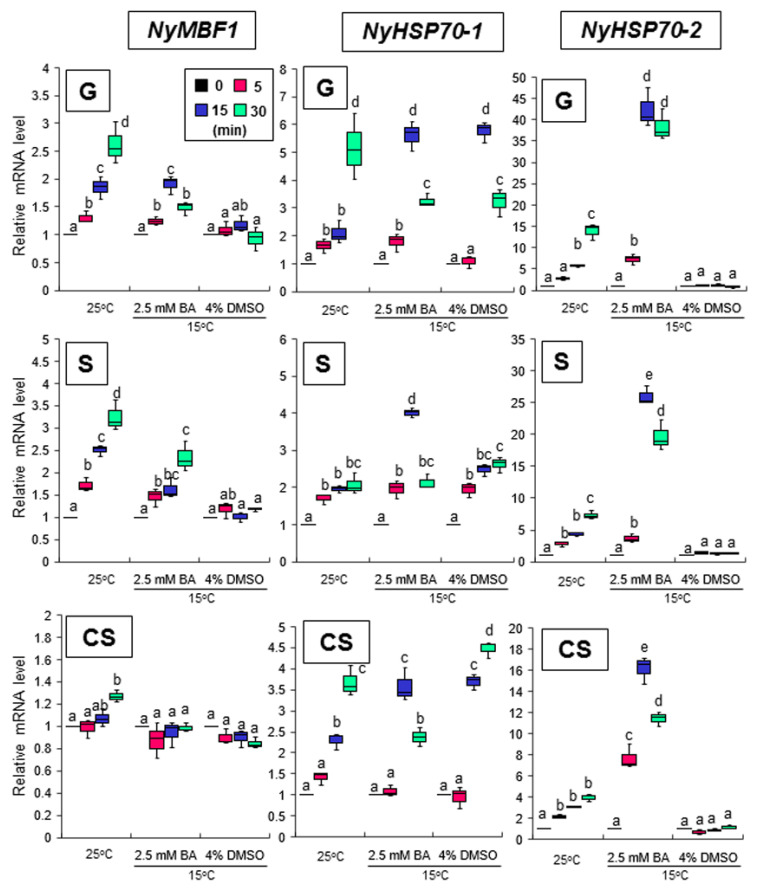
Effects of changes in membrane physical state on *NyMBF1*, *NyHSP70-1*, and *NyHSP70-2* expression in three life cycle generations of *Neopyropia yezoensis*. The algae were treated with 2.5 mM BA and 4% DMSO at 15 °C for 5, 15, and 30 min, and the expression of *NyHSP70-1* and *NyHSP70-2* was compared with their expression at 25 °C. Relative mRNA levels were normalized using *eIF4A* as the reference gene. In the box plots, values on the Y axis represent the fold change of relative quantification of each gene. Different alphabetical letters denote significant differences in expression level among the three life cycle generations from triplicate independent replicates as defined by Tukey’s test (*p* < 0.05) in two-way ANOVA. G—gametophyte; S—sporophyte; CS—conchosporophyte.

Treatment with 2.5 mM BA at 15 °C did not induce expression of *NyHTR2* or *NyHTR2L* but did (like heat stress) induce expression of *NyMBF1*, *NyHSP70-1*, and *NyHSP70-2* (Figure 5 and Figure 6). In contrast, treatment with 4% DMSO did not induce the expression of *NyHTR2*, *NyHTR2L*, *NyMBF1*, or *NyHSP70-2*, but did induce expression of *NyHSP70-1* (Figure 5 and Figure 6). This indicates that the heat-induced expression of *NyHTR2* or *NyHTR2L* is not dependent on membrane fluidization, whereas that of *NyMBF1*, *NyHSP70-1*, and *NyHSP70-2* is fluidization-dependent.

Both cold stress and DMSO rigidify membranes; however, a 5 °C exposure did not induce expression of *NyHSP70-1* (Figure S6). To further assess whether *NyHSP70-1* responds to low temperatures, we examined the expression of *NyHSP70-1* and other genes in algae exposed to a temperature of 0 °C. Only *NyHSP70-1* was induced by 0 °C treatment (Figure S7). Thus, *NyHSP70-1* is inducible by both heat and cold, whereas the other genes we examined were only inducible by heat.

**Figure 7:**
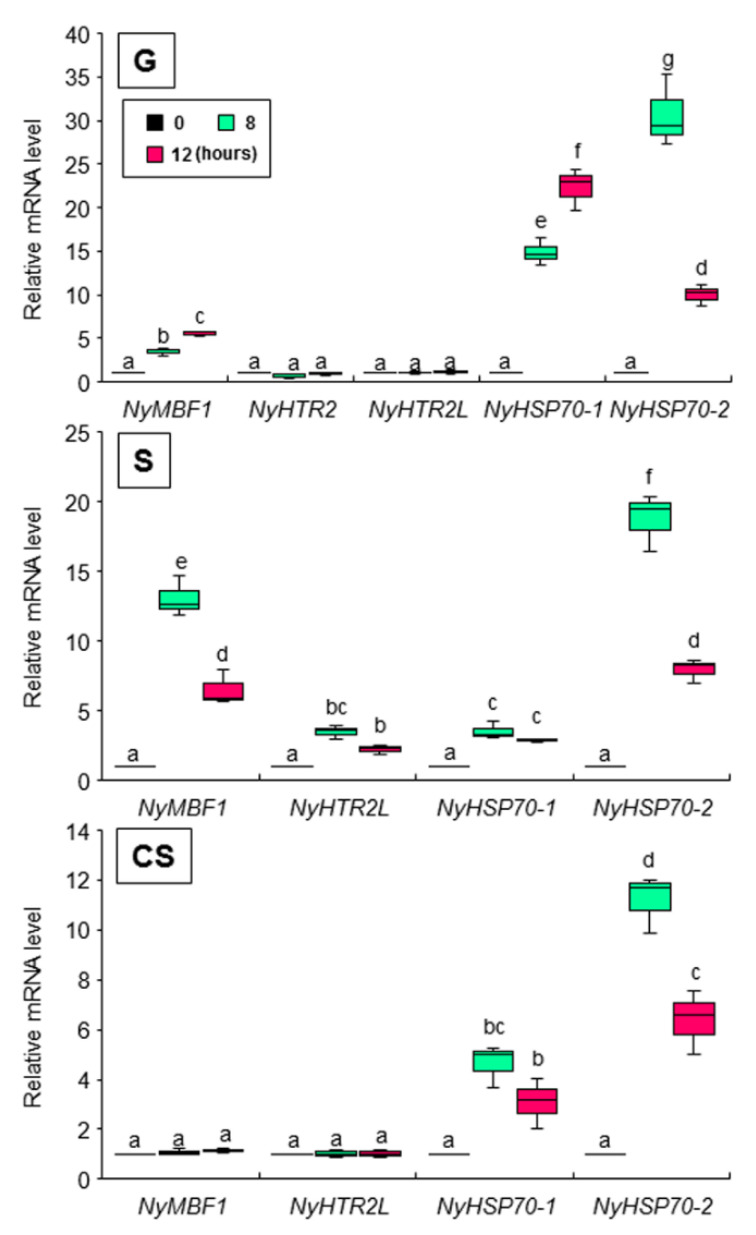
Involvement of membrane fluidization in dark-induced gene expression in three life cycle generations of *Neopyropia yezoensis*. The algae were treated with 2.5 mM BA, and expression of *NyHTR2*, *NyHTR2L*, *NyMBF1*, *NyHSP70-1*, and *NyHSP70-2* was examined at 8 and 12 h after heat stress (25 °C) exposure under continual light. Relative mRNA levels were normalized using *eIF4A* as the reference gene. In the box plots, values on the Y axis represent the fold change of relative quantification of each gene. Different alphabetical letters denote significant differences in expression level among the three life cycle generations from triplicate independent replicates as defined by Tukey’s test (*p* < 0.05) in two-way ANOVA. G—gametophyte; S—sporophyte; CS—conchosporophyte.

**Figure 8:**
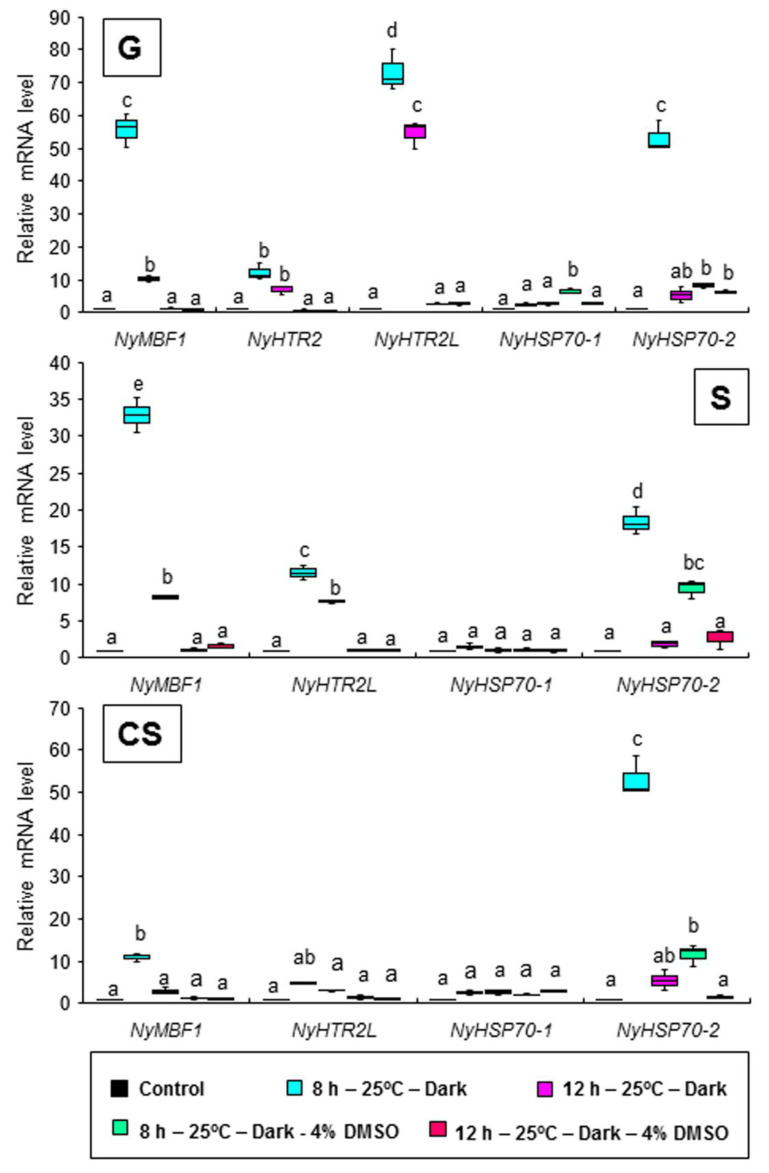
Reduction in the dark-induced second expression peak by membrane rigidification in three life cycle generations of *Neopyropia yezoensis*. The algae were treated with 4% DMSO, and expression of *NyHTR2*, *NyHTR2L*, *NyMBF1*, *NyHSP70-1*, and *NyHSP70-2* was examined 8 and 12 h after heat stress exposure (25 °C) in the dark. Relative mRNA levels were normalized using *eIF4A* as the reference gene. In the box plots, values on the Y axis represent the fold change of relative quantification of each gene. Different alphabetical letters denote significant differences in expression level among the three life cycle generations from triplicate independent replicates as defined by Tukey’s test (*p* < 0.05) in two-way ANOVA. G—gametophyte; S—sporophyte; CS—conchosporophyte.

Finally, we performed two experiments to confirm whether membrane fluidization is required for the second peak seen under dark conditions. First, we subjected the three generations of algae to 25 °C and 2.5 mM BA under continuous light. This treatment induced the expression of *NyHSP70-1* and *NyHSP70-2*, as detected 1 h after BA application, but had no effect on the expression of *NyHTR2* or *NyHTR2L* (Figure 7). BA application also induced *NyMBF1* in gametophytes but not in conchosporophytes (Figure 7). In the second experiment, we subjected the three generations of algae to heat stress, either with or without 4% DMSO, under dark conditions. In gametophytes, DMSO reduced expression levels of all genes except *NyHSP70-1* (Figure 8). This is consistent with our earlier results regarding the effect of DMSO treatment on *NyHSP70-1* under dark conditions (Figure 4 and Figure S6). Therefore, there are at least two signal transduction pathways regulating the second expression peak under dark conditions at 25 °C: a pathway involving dark-dependent membrane fluidization that governs *NyMBF1* and *NyHSP70-2* expression and another pathway that, though heat dependent, does not involve membrane fluidization and that governs *NyHTR2* and *NyHTR2L* expression.

## 4. Discussion

In the present study, we examined the involvement of membrane fluidity in heat-stress-inducible gene expression in the red alga *N. yezoensis*. We identified two genes encoding *NsHTR2* homologs in *N. yezoensis*, which we designated *NyHTR2* and *NyHTR2L* (Figure 1). The *NsHTR2* homologs contained a novel Cys-repeat motif that was conserved only in Bangiales and which we therefore designated the Bangiales Cys-repeat (BCR) motif. We identified *NyHTR2* as a gametophyte-specific gene (Figure 2). Both *NyHTR2* and *NyHTR2L* were heat-stress-inducible genes but did not depend on the membrane fluidization that generally results from heat stress, as evidenced by their lack of induction by the membrane fluidizer BA (Figure 5 and Figure 7). In contrast, expression of *NyMBF1*, *NyHSP70-1*, and *NyHSP70-2* was induced not only by heat but also by membrane fluidization (BA treatment); thus, we consider them to be membrane-fluidization-dependent expressions (Figure 7 and Figure S7). Therefore, we conclude that *N. yezoensis* has both membrane-fluidization-dependent and -independent pathways of heat-inducible expression.

We found that dark conditions stimulated both the membrane-fluidization-dependent and -independent pathways, but in different fashions. The heat-inducible genes had an initial, transient low-level expression peak at 4–6 h after heat exposure in the light (Figure 2 and Figures S3–S6). A second expression peak occurred 1 h after the dark period began; however, this did not occur when *N. yezoensis* was grown under continual light at 25 °C (Figure 3 and Figure 4). This indicates that the three generations of *N. yezoensis* could tolerate continual heat stress after the first peak; however, dark exposure reset the heat stress response and resulted in a second expression peak. Moreover, it is likely that membrane fluidization is involved in the second heat-induced expression peak of *NyMBF1* and *NyHSP70-2* because BA treatment also induced a second expression peak of these genes under continual light, whereas DMSO reduced the second expression peak in the dark period (Figure 7 and Figure 8).

In contrast, the second, dark-induced expression peaks of *NyHTR2* and *NyHTR2L* were not sensitive to BA application, indicating that they are independent of membrane fluidization. However, DMSO treatment reduced the dark-induced expression of these genes (Figure 8). DMSO is a membrane rigidizer and is often used to study physiological responses to membrane rigidification, such as cold-stress-inducible gene expression [34,49]. Therefore, the fact that DMSO reduced *NyHTR2* and *NyHTR2L* expression is inconsistent with the BA treatment results. In the unicellular green alga *Chlamydomonas reinhardtii*, transcripts of *HSF1*, *HSP70A*, *HSP90A*, and *HSP90C* accumulated under treatment with heat plus DMSO, an effect similar to that of the cytosolic translation inhibitor cycloheximide. Therefore, DMSO-dependent translational inhibition is proposed to be responsible for the enhanced expression of these genes caused by DMSO [50,51]. It is possible that translational inhibition by DMSO is also related to *NyHTR2* and *NyHTR2L* expression in *N. yezoensis*; however, this remains to be confirmed.

On the basis of our findings, we conclude that there are multiple pathways regulating heat-stress-inducible gene expression in *N. yezoensis*. One is a membrane-fluidization-dependent pathway, which can be subdivided into a dark-enhanced pathway for *NyMBF1* and *NyHSP70-2* and a dark-independent pathway for *NyHSP70-1*. Similarly, moss membrane proteins, including the cyclic nucleotide-gated Ca^2+^ channels CNGCb and CNGCd, are membrane-fluidization-dependent heat-sensing molecules [29,36,52]. Therefore, it could be valuable to explore the involvement of Ca^2+^ influx and cyclic nucleotide-gated Ca^2+^ channels in early heat stress responses in *N. yezoensis*. The other pathway regulating heat-stress-inducible gene expression in *N. yezoensis* is a membrane-fluidization-independent pathway directing *NyHTR2* and *NyHTR2L* expression, which is enhanced by dark conditions. The discovery of this pathway was unexpected because heat stress responses are generally thought to be triggered by membrane fluidization [30,31,32,33,52]. Thus, further examination of heat-stress-inducible expression of *NyHTR2* and *NyHTR2L* might uncover novel mechanisms of heat stress responses in photosynthetic organisms.

Alone among the genes examined in this study, *NyHSP70-1* was induced by both DMSO treatment and cold stress (Figure 6 and Figure S7), indicating that it is triggered by cold-stress-induced membrane rigidification. Similar cold-stress-inducible expression of *HSP* genes has been reported in various plant species [41,53,54,55,56,57,58]. In our study, *NyHSP70-1* expression was also induced by heat stress and was membrane-fluidization-dependent, but it was not affected by dark (Figure 4, Figure 6, Figure 7 and Figure 8, and Figure S6). *NyHSP70-1* is therefore an ideal model in which to investigate the regulatory mechanisms of heat and cold-stress-inducible gene expression based on physical changes in membranes in Bangiales.

Extracellular Ca^2+^ influxes and reactive oxygen species (ROS) are involved in the early phases of heat stress responses in terrestrial plants [59,60,61,62,63]. These can be triggered by membrane fluidization [34,35,36,52,64]. However, little is known about how these signaling molecules may be involved in either of the heat-stress-induced gene expression pathways in *N. yezoensis*. Therefore, further examination of the membrane-fluidization-dependent and -independent pathways could provide novel insights into stress perception and early events in heat-stress-inducible gene expression in Bangiales.

## Data Availability

Data are contained within this article or Supplementary Materials.

## Supplementary Materials

The following supporting information can be downloaded at: https://www.mdpi.com/article/10.3390/cells11091486/s1, Figure S1: Conservation of the Bangiales cysteine-rich (BCR) motif in HTR2 homologs from *Neopyropia yezoensis*, *Neopyropia seriata*, and *Porphyra umbilicalis*; Figure S2: Cycle threshold (Ct) values and gene expression stability values (*M*) of candidate reference genes in three life cycle generations of *Neopyropia yezoensis*; Figure S3: Temperature-dependent expression patterns of *NyMBF1* in *Neopyropia yezoensis* gametophytes; Figure S4: Temperature-dependent expression patterns of *NyMBF1* in *Neopyropia yezoensis* sporophytes; Figure S5: Temperature-dependent expression patterns of *NyMBF1* in *Neopyropia yezoensis* conchosporophytes; Figure S6: Temperature-dependent expression patterns of *NyHSP70-1* and *NyHSP70-2* with temporal comparisons in three life cycle generations of *Neopyropia yezoensis*; Figure S7: Induction of *NyHSP70-1* by exposure to 0 °C in three life cycle generations of *Neopyropia yezoensis*; Table S1: Sequences of primers for candidates of reference genes used for qRT-PCR; Table S2: Expression stability analysis of four reference genes by NormFinder in three life cycle generations of *Neopyropia yezoensis*; Table S3: Sequences of primers for heat stress-inducible genes used for qRT-PCR; Table S4: Temperature-dependent gene expression profiles of *NyMBF1* in three life cycle generations of *Neopyropia yezoensis* normalized using reference genes; Table S5: Gene expression profiles of *NyHTR2* and *NyHTR2L* in three generations of *Neopyropia yezoensis* under heat stress normalized using *eIF4A* as the reference gene; Table S6: Gene expression profiles of *NyHSP70-1* and *NyHSP70-2* in three generations of *Neopyropia yezoensis* under heat stress normalized using *eIF4A* as the reference gene.
