# Supplementary material for: Membrane-Fluidization-Dependent and -Independent Pathways Are Involved in Heat-Stress-Inducible Gene Expression in the Marine Red Alga Neopyropia yezoensis
Source: Cells. 2022 Apr 28;11(9):1486. doi: 10.3390/cells11091486 (PMC9100149; doi:10.3390/cells11091486)
Supplement: Supplementary file 1 [file Figure S1.pdf › Supplementary files for submission/Figure S1.pdf]

```
NyHTR2     -------------------------------------------------------------
NyHTR2L    MPSGGRVAAAATRSPDQYKSSVTRPMVGGGECRPPTIPLRATSNQRRGNSPPFRDSPFPLS 61
NsHTR2     -------------------------------------------------------------
OSX76372   -------------------------------------------------------------
OSX78858   -------------------------------------------------------------
OSX74332   -------------------------------------------------------------
OSX71170   -------------------------------------------------------------
OSX80752   -------------------------------------------------------------
OSX79754   -------------------------------------------------------------
OSX68660   -------------------------------------------------------------
OSX76191   -------------------------------------------------------------

NyHTR2     ------------------MALSKTRFAALAVAVMTLLTAAAAAPAADAAAAAA-------- 35
NyHTR2L    REHPPSPYPPRPPTSIVDMALSKLLMV---TALLAMLALPMASAA---------------- 103
NsHTR2     ------------------MAASKTLAAAFAVALLALLTLTAAAPAADTA--AA-------- 33
OSX76372   ------------------MAVPKSLFASLAVALLALLSVAAVSPTDAAV---A-------- 32
OSX78858   ------------------MAVPKSLFASLAVALLALLSVAAVSPADAAV---A-------- 32
OSX74332   ------------------MAVPKSLFASLAVALLALLSVAAVSPADAAV---A-------- 32
OSX71170   ------------------MVVSKSLFASLAVALLALLSVAAVSPADAAV---A-------- 32
OSX80752   ------------------MGAIKMLLVTAIVASLALLTVAASRPVAADDSADDMVDGAWPT 43
OSX79754   ------------------MAATKFLAL--LLTFVALLSVVAA-TD---------------- 24
OSX68660   ------------------MAATKALAL--LLTLVALVSLATA-TA---------------- 24
OSX76191   ------------------MAR-------FTLPLLVLLVAALV-PLVASAS----------- 24

NyHTR2     -DAATVEYESLRIEGLSDVADAPALTGLADRSPYGGGYGCRAYFKCQQKTEKVVAKHTYAC 95
NyHTR2L    ---LPV-----DREAVSAVADAPGLSAL--VR---ATEAC-SYFQCGSPTVEVVAKHTYPC 150
NsHTR2     -DAATAAYDALHVEGISGAAEAPALDGLTDRSG---YYRCRSYFVCGSKKVKVPVKHSHPC 90
OSX76372   -VQAASNPEALHIEGLSHADEEPALASLTGR-TR-YEGRCNSYFKCGTKSVTVVAKNSYLC 90
OSX78858   -VQAAPDPEALHIEGLSRADEEPALASLTVRS---PYGVCKGYFQCGTKSVTVEAKNTYLC 89
OSX74332   -VQAASDPEALHIEGLSRADEEPALASLTDRTVR-YGGICNRYFKCGTKSVTVVAKNSYLC 91
OSX71170   -VQAASDPEALHIEGLSRADEEPALASLTAHSYYGGHDTCNGYFKCGKKSVYVVAKASYLC 92
OSX80752   GADDDVPADVVVIGGHTPLNEAPGLADITARQQV-VAKKC-FYFKCNTKRVRRVVRQAWRC 102
OSX79754   ---VPPELEEEPVIGTSRVDAAPGLADITAVH----WGRCSSYFRCGRKLIFPKVKATYKC 78
OSX68660   ---VPAQ--GEDVFGSSTLIDAPGLSDIAAVR----ARKCSSYFPCGWRTATGQAKVSYKC 76
OSX76191   -----PA--SDVLSSVARTHGGPHDVSTVK------AGTCGGYVKCGKNTVKNTVKNSRYC 72

                              BCR motif
NyHTR2     YFKIASSAA--ARSAGGLCRPNPYGCV--AAKCGGWVKCKCDKCQTVSITYPVWCEQ     148
NyHTR2L    WFKVATGN---KGLAPDTCSPTPYDCVD-DATCKGWKQCKCDVCKTVKLTKKEYCKLPEE  206
NsHTR2     YYKEAPSIL-KSSRSSGLCRPDPYSCK--PATCIGWRKCTCDACKTVIFTHNIWCEK     144
OSX76372   YFKEAPSKA--SRSGSGRCNGLPYKCK--PAKCTGWRVCTCDQCKKVVFKVPIYCEK     143
OSX78858   YYKEAKSRS--T--RSSSCNTLPYNCK--PATCTGWRVCKCDQCKRVTFTFPIYCEQ     140
OSX74332   YYKEASYKI--GASGSGSCSSLPYNCK--RAKCTGWRVCTCNHCKRVTFTIPIYCEK     144
OSX71170   YFKEAESYR--GKAYSGECSSLPYNCK--PAKCPGWRKCTCDKCKKVLIKVPIYCEL     145
OSX80752   FFKVAFKITPKPKPTGSLCHPTPYACT--KSACGGWRRCLCDACKVVVFTYPVWCMKK    158
OSX79754   YFRTAASA--LPAKGPKRCGRFPYNCK--NAHCKGWKVCTCTKCFRARTRVPQWCEHP    132
OSX68660   YFKVAASLA-RPEAAKNGCAPHPYKCR--DAKCKGWKQCTCDKCFSAKTKVRVWCEHP    131
OSX76191   YFKLKTKPSPAKGDSKRNCKRAPYSCKRVASPCNGWYKCKCDECSTVKTEVDRWCLKK    130
```

Figure S1. Conservation of the Bangiales cysteine-rich (BCR) motif in HTR2 homologs from *Neopyropia yezoensis*, *Neopyropia seriata*, and *Porphyra umbilicalis*. Cysteine residues and amino acids conserved in all 11 proteins are highlighted in black and dark blue (with white characters), respectively. Yellow backgrounds indicate amino acids conserved in 7 to 10 proteins. The position of the BCR motif is indicated by a red bar. The amino acid position numbers are indicated on the right.
