# Supplementary material for: Membrane-Fluidization-Dependent and -Independent Pathways Are Involved in Heat-Stress-Inducible Gene Expression in the Marine Red Alga Neopyropia yezoensis
Source: Cells. 2022 Apr 28;11(9):1486. doi: 10.3390/cells11091486 (PMC9100149; doi:10.3390/cells11091486)
Supplement: Supplementary file 1 [file cells-11-01486-s001.zip › Supplementary files for submission/Figure S2.pdf]

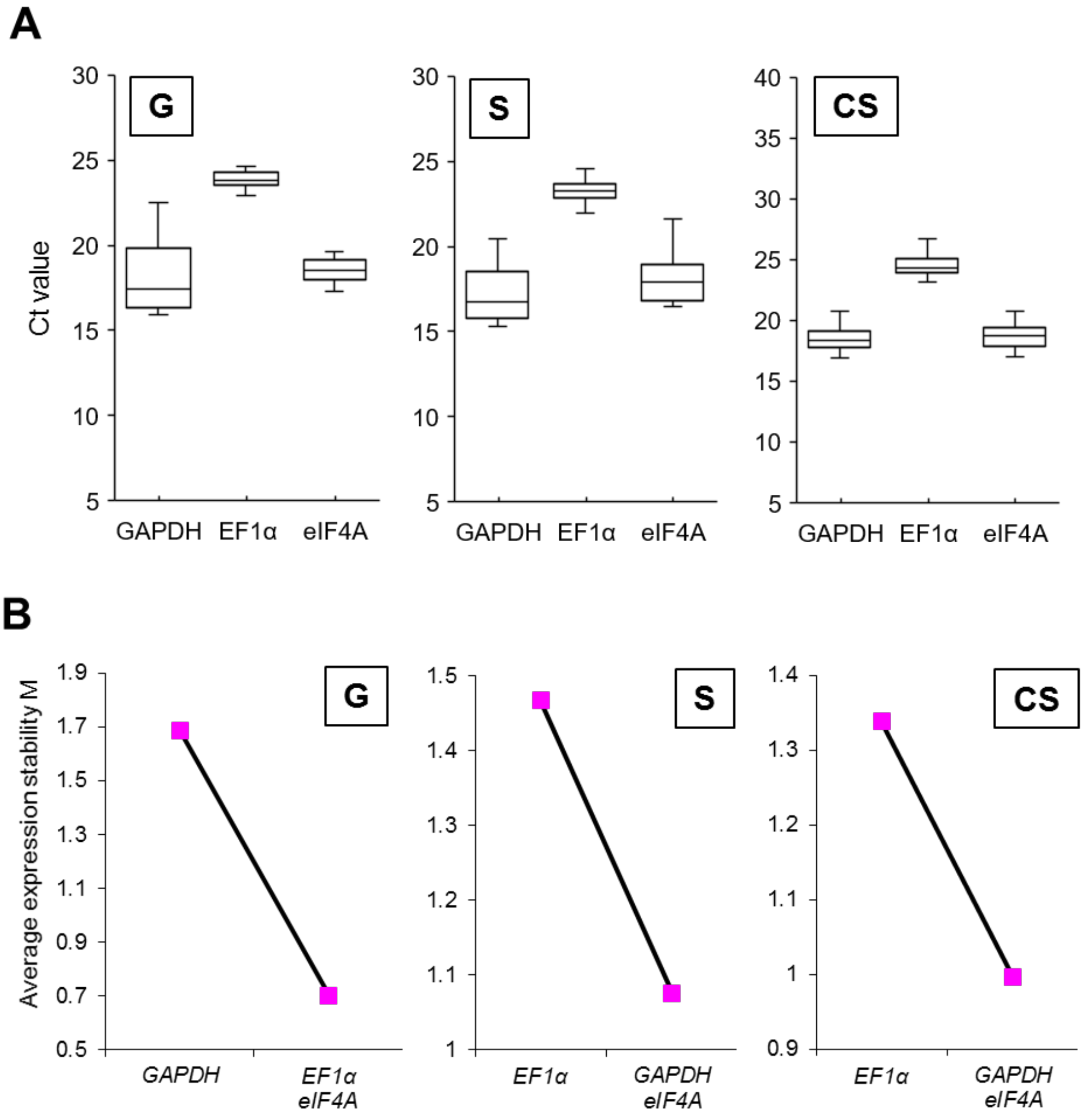


Figure S2. Cycle threshold (Ct) values and gene expression stability values (*M*) of candidate reference genes in three life cycle generations of *Neopyropia yezoensis*. (A) Ct values of candidate reference genes. Boxes show the median (center lines), Q1 (lower outline), Q3 (upper outline), and 1.5 times the interquartile range above Q3 and below Q1 (whiskers). Data were derived from 3 replicates of 27 culture samples. (B) *M* was calculated by stepwise exclusion of the least stable gene in three generations using geNorm. In each plot, the least stable gene is on the left and the most stable gene is on the right. Data were derived from 3 replicates of 22 culture samples for each generation. G, gametophyte; S, sporophyte; CS, conchosporophyte.
