# Supplementary material for: Membrane-Fluidization-Dependent and -Independent Pathways Are Involved in Heat-Stress-Inducible Gene Expression in the Marine Red Alga Neopyropia yezoensis
Source: Cells. 2022 Apr 28;11(9):1486. doi: 10.3390/cells11091486 (PMC9100149; doi:10.3390/cells11091486)
Supplement: Supplementary file 1 [file cells-11-01486-s001.zip › Supplementary files for submission/Figure S5.pdf]

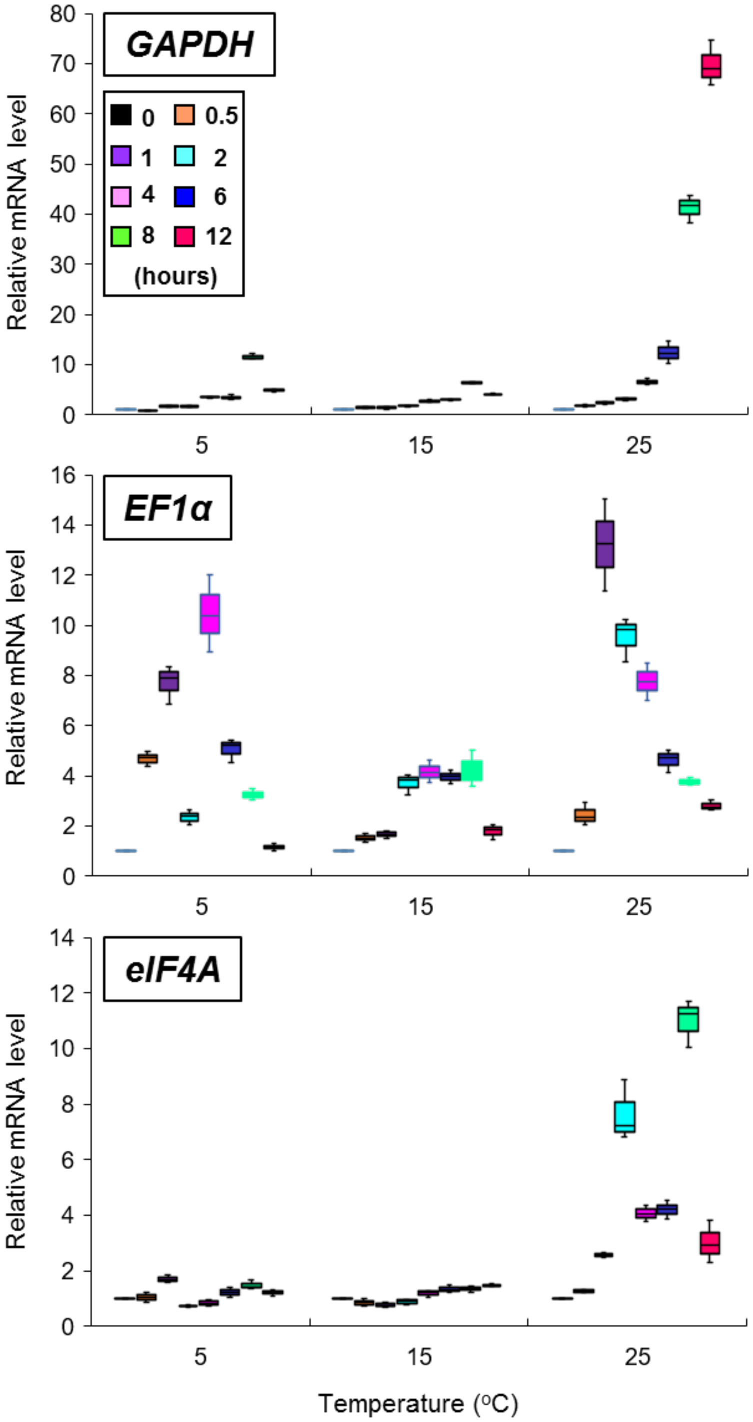

Figure S5. Temperature-dependent expression patterns of *NyMBF1* in *Neopyropia yezoensis* conchosporophytes. Relative mRNA levels at 5, 15, and 25°C were normalized using *GAPDH*, *EF1α*, or *eIF4A* as the reference gene. Significant differences in expression level among the three life cycle generations from triplicate independent replicates as defined by Tukey's test ($p < 0.05$) in two-way ANOVA are shown in Table S4.
