# Supplementary material for: Membrane-Fluidization-Dependent and -Independent Pathways Are Involved in Heat-Stress-Inducible Gene Expression in the Marine Red Alga Neopyropia yezoensis
Source: Cells. 2022 Apr 28;11(9):1486. doi: 10.3390/cells11091486 (PMC9100149; doi:10.3390/cells11091486)
Supplement: Supplementary file 1 [file cells-11-01486-s001.zip › Supplementary files for submission/Figure S7.pdf]

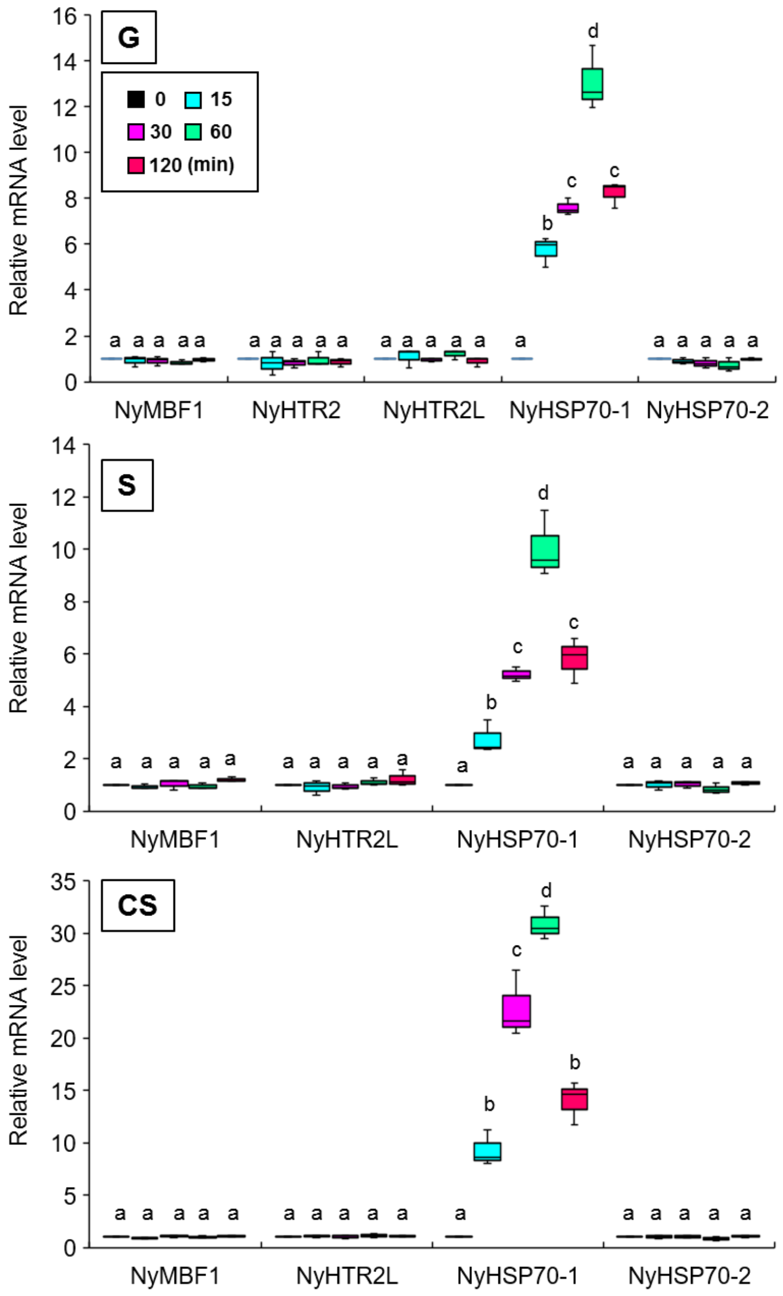

Figure S7. Induction of *NyHSP70-1* by exposure to 0°C in three life cycle generations of *Neopyropia yezoensis*. Generations were incubated at 0°C for 15, 30, 60, and 120 min, and expression of *NyHTR2*, *NyHTR2L*, *NyMBF1*, *NyHSP70-1*, and *NyHSP70-2* was examined. Relative mRNA levels were normalized using *eIF4A* as the reference gene. In the box plots, values on the Y axis represent the fold change of relative quantification of each gene. Different letters denote significant differences in expression level among the three life cycle generations from triplicate independent replicates as defined by Tukey's test ($p < 0.05$) in two-way ANOVA. G, gametophyte; S, sporophyte; CS, conchosporophyte.
