# Supplementary material for: Membrane-Fluidization-Dependent and -Independent Pathways Are Involved in Heat-Stress-Inducible Gene Expression in the Marine Red Alga Neopyropia yezoensis
Source: Cells. 2022 Apr 28;11(9):1486. doi: 10.3390/cells11091486 (PMC9100149; doi:10.3390/cells11091486)
Supplement: Supplementary file 1 [file cells-11-01486-s001.zip › Supplementary files for submission/Table S1.pdf]

Table S1. Sequences of primers for candidates of reference genes used for qRT-PCR

Gene	Primer sequence (5' to 3')	Amplicon length (bp)	Efficiency (%)	Correlation coefficient (R^2)
GAPDH	CCAACAAGTGGGAGTAAGCG<br>GGACAGAACCGAACAGCGTA	104	99.1	0.997
EF1α	TTTTGGTGGCACTGCTGAT<br>TACACGCACAAGTTGGGGA	141	98	0.9975
eIF4A	GCTTTCTGTCTGGACGAGG<br>TCTTCACAAGGATGCGGAT	181	101	0.9992
