# Supplementary material for: Membrane-Fluidization-Dependent and -Independent Pathways Are Involved in Heat-Stress-Inducible Gene Expression in the Marine Red Alga Neopyropia yezoensis
Source: Cells. 2022 Apr 28;11(9):1486. doi: 10.3390/cells11091486 (PMC9100149; doi:10.3390/cells11091486)
Supplement: Supplementary file 1 [file cells-11-01486-s001.zip › Supplementary files for submission/Table S2.pdf]

Table S2. Expression stability analysis of four reference genes by NormFinder in three life cycle generations of *Neopyropia yezoensis*

Gametophyte			Sporophyte			Conchosporophyte		
**Rank**	**Gene**	**Stability value**	**Rank**	**Gene**	**Stability value**	**Rank**	**Gene**	**Stability value**
1	*eIF4A*	0.137	1	*eIF4A*	0.278	1	*eIF4A*	0.134
2	*EF1α*	0.208	2	*GAPDH*	0.348	2	*GAPDH*	0.300
3	*GAPDH*	0.544	3	*EF1α*	0.403	3	*EF1α*	0.326
