# Supplementary material for: Membrane-Fluidization-Dependent and -Independent Pathways Are Involved in Heat-Stress-Inducible Gene Expression in the Marine Red Alga Neopyropia yezoensis
Source: Cells. 2022 Apr 28;11(9):1486. doi: 10.3390/cells11091486 (PMC9100149; doi:10.3390/cells11091486)
Supplement: Supplementary file 1 [file cells-11-01486-s001.zip › Supplementary files for submission/Table S3.pdf]

Table S3. Sequences of primers for heat stress-inducible genes used for qRT-PCR

Primer name	Primer sequence (5' to 3')	Annealing temperature (°C)	Product size (bp)
Q-NyMBF1-F	TCGAGCTTGGAGAGATTGGT	58	114
Q-NyMBF1-R	GAAGAAGGCGGATGTCTCTG		
Q-NyHTR2-F	CACACCTACGCGTGCTACTT	59	134
Q-NyHTR2-R	CACTTGTCGCACTTGCACTT		
Q-NyHTR2L-F	CATACCTACCCGTGCTGGTT	60	134
Q-NyHTR2L-R	CACACGTCACACTTGCACTG		
Q-NyHSP70-1-F	GGTTCGCACCCCAACATCAC	59	112
Q-NyHSP70-1-R	TCACAGCCACAACCTCTCACC		
Q-NyHSP70-2-F	CATTGGTGACTCAGCGAAGA	59	143
Q-NyHSP70-2-R	CCTTCTGCACCACCTTGAAT		
