# Supplementary material for: Membrane-Fluidization-Dependent and -Independent Pathways Are Involved in Heat-Stress-Inducible Gene Expression in the Marine Red Alga Neopyropia yezoensis
Source: Cells. 2022 Apr 28;11(9):1486. doi: 10.3390/cells11091486 (PMC9100149; doi:10.3390/cells11091486)
Supplement: Supplementary file 1 [file cells-11-01486-s001.zip › Supplementary files for submission/Table S4.pdf]

Table S4. Temperature-dependent gene expression profiles of *NyMBF1* in three life cycle generations of *Neopyropia yezoensis* normalized using reference genes

Generation	Reference gene	Temperature	Duration (h)							
			0	0.5	1	2	4	6	8	12
G	*GAPDH*	5°C	1 ± 0^{a}	1.32 ± 0.07^{a}	1.85 ± 0.09^{a}	2.09 ± 0.04^{a}	1.49 ± 0.04^{a}	3.71 ± 0.13^{a}	6.3 ± 0.97^{ab}	20.2 ± 2.55^{c}
		15°C	1 ± 0^{a}	0.76 ± 0.08^{a}	1.11 ± 0.13^{a}	1.71 ± 0.16^{a}	10.92 ± 1.37^{b}	24.75 ± 1.75^{cd}	136.98 ± 2.85^{f}	21.38 ± 1.88^{cd}
		25°C	1 ± 0^{a}	1.11 ± 0.12^{a}	2.56 ± 0.1^{a}	4.87 ± 0.11^{ab}	26.35 ± 2.06^{d}	41.86 ± 3.01^{e}	143.54 ± 6.27^{g}	20.05 ± 2.07^{c}
	EF1α	5°C	1 ± 0^{a}	2.75 ± 0.08^{g}	3.95 ± 0.18^{j}	2.33 ± 0.17^{ef}	1.28 ± 0.22^{ab}	2.03 ± 0.19^{cde}	1.18 ± 0.17^{a}	1.12 ± 0.13^{a}
		15°C	1 ± 0^{a}	1.32 ± 0.09^{ab}	2.26 ± 0.17^{def}	1.67 ± 0.2^{bc}	1.65 ± 0.2^{bc}	1.77 ± 0.19^{c}	1.87 ± 0.18^{cd}	1.06 ± 0.16^{a}
		25°C	1 ± 0^{a}	1.74 ± 0.15^{c}	2.5 ± 0.14^{fg}	3.54 ± 0.12^{ij}	6.27 ± 0.22^{l}	3.22 ± 0.23^{i}	4.7 ± 0.41^{k}	1.07 ± 0.06^{a}
	eIF4A	5°C	1 ± 0^{a}	1.9 ± 0.59^{a}	1.57 ± 0.12^{ab}	1.64 ± 0.12^{ab}	1.85 ± 0.18^{ab}	1.4 ± 0.25^{a}	1.65 ± 0.26^{a}	3.44 ± 0.1^{ab}
		15°C	1 ± 0^{a}	2.2 ± 0.18^{ab}	1.92 ± 0.14^{b}	2.89 ± 0.15^{ab}	3.15 ± 0.1^{ab}	3.2 ± 0.15^{ab}	3.71 ± 0.27^{ab}	3.26 ± 0.22^{ab}
		25°C	1 ± 0^{a}	2.61 ± 0.37^{ab}	6.15 ± 0.14^{bc}	43.27 ± 3.58^{f}	19.07 ± 1.6^{e}	10.94 ± 1.7^{d}	55.72 ± 5.15^{g}	10.4 ± 1^{cd}
S	*GAPDH*	5°C	1 ± 0^{a}	1.05 ± 0.27^{a}	1.07 ± 0.15^{a}	1.15 ± 0.13^{a}	1.51 ± 0.16^{a}	2.55 ± 0.25^{ab}	5.23 ± 0.79^{cd}	9.44 ± 1.18^{ef}
		15°C	1 ± 0^{a}	0.97 ± 0.09^{a}	1.23 ± 0.15^{a}	1.47 ± 0.23^{ab}	3.25 ± 0.34^{abc}	7.57 ± 0.34^{de}	7.18 ± 0.86^{de}	2.43 ± 0.15^{ab}
		25°C	1 ± 0^{a}	2.15 ± 0.2^{ab}	4.48 ± 0.42^{bc}	8.03 ± 0.31^{e}	10.88 ± 0.66^{f}	18.68 ± 2.05^{g}	17.74 ± 1.84^{g}	7.71 ± 1.09^{e}
	EF1α	5°C	1 ± 0^{a}	1.54 ± 0.22^{a}	2.99 ± 0.28^{ab}	3.31 ± 0.27^{ab}	3.12 ± 0.23^{ab}	1.34 ± 0.2^{a}	1.41 ± 0.15^{a}	1.05 ± 0.18^{a}
		15°C	1 ± 0^{a}	1.01 ± 0.23^{a}	7.48 ± 0.51^{de}	5.66 ± 0.49^{bcd}	3.59 ± 0.49^{abc}	2.39 ± 0.22^{a}	1.32 ± 0.07^{a}	1.15 ± 0.17^{a}
		25°C	1 ± 0^{a}	2.89 ± 0.36^{ab}	32.13 ± 4.36^{g}	12.52 ± 0.47^{f}	10.19 ± 1.09^{ef}	6.6 ± 0.43^{cd}	2.45 ± 0.37^{ab}	1.43 ± 0.22^{a}
	eIF4A	5°C	1 ± 0^{a}	0.84 ± 0.08^{a}	0.96 ± 0.08^{a}	0.93 ± 0.09^{a}	1 ± 0.04^{a}	0.8 ± 0.15^{a}	0.98 ± 0.24^{a}	2.41 ± 0.2^{a}
		15°C	1 ± 0^{a}	1.08 ± 0.03^{a}	1.61 ± 0.37^{a}	1.92 ± 0.32^{a}	2.15 ± 0.2^{a}	2.62 ± 0.15^{a}	2.23 ± 0.18^{a}	2.34 ± 0.17^{a}
		25°C	1 ± 0^{a}	3.24 ± 0.35^{a}	10.28 ± 1.21^{bc}	33.57 ± 2.84^{e}	13.28 ± 1.99^{cd}	13.86 ± 1.51^{d}	32.86 ± 2.29^{e}	8.21 ± 0.26^{b}
CS	*GAPDH*	5°C	1 ± 0^{ab}	0.86 ± 0.07^{a}	1.76 ± 0.2^{ab}	1.74 ± 0.07^{ab}	3.54 ± 0.11^{abc}	3.52 ± 0.45^{abc}	11.61 ± 0.58^{d}	4.93 ± 0.32^{bc}
		15°C	1 ± 0^{ab}	1.52 ± 0.15^{ab}	1.38 ± 0.28^{ab}	1.79 ± 0.1^{ab}	2.79 ± 0.16^{abc}	3.03 ± 0.08^{abc}	6.48 ± 0.13^{c}	4.13 ± 0.12^{abc}
		25°C	1 ± 0^{ab}	1.85 ± 0.15^{ab}	2.4 ± 0.27^{ab}	3.14 ± 0.16^{abc}	6.61 ± 0.65^{c}	12.51 ± 2.23^{d}	41.25 ± 2.69^{e}	69.79 ± 4.62^{f}
	EF1α	5°C	1 ± 0^{a}	4.69 ± 0.3^{ef}	7.71 ± 0.77^{g}	2.36 ± 0.31^{bcd}	10.47 ± 1.53^{h}	5.07 ± 0.47^{f}	3.25 ± 0.23^{cdef}	1.16 ± 0.14^{ab}
		15°C	1 ± 0^{a}	1.53 ± 0.18^{ab}	1.67 ± 0.15^{abc}	3.7 ± 0.41^{def}	4.18 ± 0.44^{def}	3.97 ± 0.26^{def}	4.24 ± 0.73^{def}	1.79 ± 0.3^{abc}
		25°C	1 ± 0^{a}	2.45 ± 0.5^{abcd}	13.21 ± 1.84^{i}	9.54 ± 0.87^{gh}	7.77 ± 0.73^{g}	4.62 ± 0.46^{ef}	3.77 ± 0.17^{def}	2.8 ± 0.19^{bcde}
	eIF4A	5°C	1 ± 0^{a}	1.06 ± 0.18^{a}	1.69 ± 0.15^{ab}	0.73 ± 0.03^{a}	0.84 ± 0.11^{a}	1.21 ± 0.19^{a}	1.48 ± 0.16^{ab}	1.22 ± 0.11^{a}
		15°C	1 ± 0^{a}	0.86 ± 0.13^{a}	0.77 ± 0.09^{a}	0.87 ± 0.11^{a}	1.18 ± 0.13^{a}	1.34 ± 0.13^{a}	1.34 ± 0.11^{a}	1.48 ± 0.03^{ab}
		25°C	1 ± 0^{a}	1.27 ± 0.05^{a}	2.55 ± 0.09^{bc}	7.63 ± 1.12^{f}	4.06 ± 0.29^{de}	4.2 ± 0.34^{e}	10.99 ± 0.85^{g}	3 ± 0.75^{cd}

Mean values ± SD were calculated from triplicate experiments, and letters denote statistically significant differences ($p < 0.05$) determined by two-way ANOVA. G, gametophyte; S, sporophyte; CS, conchosporophyte.
