# Supplementary material for: Membrane-Fluidization-Dependent and -Independent Pathways Are Involved in Heat-Stress-Inducible Gene Expression in the Marine Red Alga Neopyropia yezoensis
Source: Cells. 2022 Apr 28;11(9):1486. doi: 10.3390/cells11091486 (PMC9100149; doi:10.3390/cells11091486)
Supplement: Supplementary file 1 [file cells-11-01486-s001.zip › Supplementary files for submission/Table S5.pdf]

Table S5. Gene expression profiles of *NyHTR2* and *NyHTR2L* in three generations of *Neopyropia yezoensis* under heat stress normalized using *eIF4A* as the reference gene

Gene	Generation	Temperature	Duration (h)							
			0	0.5	1	2	4	6	8	12
NyHTR2	G	5°C	1 ± 0^{b}	0.03 ± 0.01^{a}	0.07 ± 0.02^{a}	0.28 ± 0.05^{a}	0.6 ± 0.2^{ab}	0.67 ± 0.06^{ab}	0.86 ± 0.09^{ab}	2.67 ± 0.45^{d}
		15°C	1 ± 0^{b}	1.47 ± 0.21^{bc}	0.94 ± 0.25^{b}	0.81 ± 0.28^{b}	1.21 ± 0.16^{bc}	1.06 ± 0.38^{bc}	1.3 ± 0.28^{bc}	1.43 ± 0.3^{c}
		25°C	1 ± 0^{b}	2.27 ± 0.62^{d}	3.63 ± 0.94^{de}	12.46 ± 1.97^{g}	3.71 ± 1.19^{de}	4.24 ± 1.02^{e}	12.19 ± 2.55^{g}	6.96 ± 1.44^{f}
NyHTR2L	G	5°C	1 ± 0^{ab}	0.07 ± 0.02^{a}	0.51 ± 0.04^{a}	2.99 ± 0.15^{ab}	2.77 ± 0.43^{ab}	0.9 ± 0.17^{ab}	0.01 ± 0^{a}	5.11 ± 0.49^{b}
		15°C	1 ± 0^{ab}	2.33 ± 0.95^{ab}	2.35 ± 1^{ab}	4.93 ± 0.35^{b}	5.43 ± 0.4^{b}	3.22 ± 0.84^{b}	1.48 ± 0.08^{ab}	4.14 ± 0.94^{b}
		25°C	1 ± 0^{ab}	8.05 ± 1.21^{c}	35.21 ± 1.52^{e}	64.43 ± 4.05^{fg}	16.87 ± 1.29^{d}	27.82 ± 5.89^{e}	73.19 ± 6.37^{g}	54.74 ± 4.37^{f}
	S	5°C	1 ± 0^{b}	1 ± 0.1^{b}	0.97 ± 0.1^{b}	0.88 ± 0.08^{b}	1.37 ± 0.02^{b}	0.51 ± 0.09^{a}	0.42 ± 0.11^{a}	0.98 ± 0.05^{b}
		15°C	1 ± 0^{b}	1.43 ± 0.08^{bc}	1.36 ± 0.32^{bc}	1.59 ± 0.44^{bc}	1.42 ± 0.15^{bc}	1.75 ± 0.11^{bc}	1.92 ± 0.2b^{c}	2.05 ± 0.3^{c}
		25°C	1 ± 0^{b}	1.53 ± 0.16^{bc}	4.11 ± 0.57^{d}	7.04 ± 0.82^{e}	1.54 ± 0.28^{bc}	4.2 ± 0.53^{d}	11.54 ± 0.97^{f}	7.74 ± 0.23^{e}
	CS	5°C	1 ± 0^{b}	0.73 ± 0.08^{ab}	1.29 ± 0.16^{bc}	0.5 ± 0.02^{ab}	0.35 ± 0.03^{a}	0.59 ± 0.06^{ab}	0.91 ± 0.1^{b}	0.93 ± 0.08^{b}
		15°C	1 ± 0^{b}	0.48 ± 0.08^{a}	0.35 ± 0.04^{a}	0.48 ± 0.05^{a}	0.71 ± 0.01^{ab}	0.64 ± 0.06^{ab}	0.62 ± 0.02^{ab}	1.04 ± 0.03^{bc}
		25°C	1 ± 0^{b}	0.64 ± 0.05^{ab}	1.41 ± 0.03^{c}	2.6 ± 0.46^{d}	1.66 ± 0.05^{cd}	1.29 ± 0.13^{bc}	4.93 ± 0.19^{e}	3.14 ± 0.24^{d}

Mean values ± SD were calculated from triplicate experiments, and letters denote statistically significant differences ($p < 0.05$) determined by two-way ANOVA. G, gametophyte; S, sporophyte; CS, conchosporophyte.
