# Supplementary material for: Membrane-Fluidization-Dependent and -Independent Pathways Are Involved in Heat-Stress-Inducible Gene Expression in the Marine Red Alga Neopyropia yezoensis
Source: Cells. 2022 Apr 28;11(9):1486. doi: 10.3390/cells11091486 (PMC9100149; doi:10.3390/cells11091486)
Supplement: Supplementary file 1 [file cells-11-01486-s001.zip › Supplementary files for submission/Table S6.pdf]

Table S6. Gene expression profiles of *NyHSP70-1* and *NyHSP70-2* in three generations of *Neopyropia yezoensis* under heat stress normalized using *eIF4A* as the reference gene

Gene	Generation	Temperature	Duration (h)							
			0	0.5	1	2	4	6	8	12
NyHSP70-1	G	5°C	1 ± 0^{a}	0.66 ± 0.08^{a}	0.59 ± 0.38^{a}	0.63 ± 0.44^{a}	0.72 ± 0.33^{a}	0.62 ± 0.19^{a}	0.75 ± 0.35^{a}	0.65 ± 0.52^{a}
		15°C	1 ± 0^{a}	0.61 ± 0.2^{a}	0.95 ± 0.57^{a}	0.55 ± 0.15^{a}	0.61 ± 0.16^{a}	0.68 ± 0.25^{a}	0.93 ± 0.41^{a}	0.86 ± 0.57^{a}
		25°C	1 ± 0^{a}	3.71 ± 1.55^{bc}	4.22 ± 0.79^{c}	13.45 ± 1.99^{e}	6.35 ± 0.93^{d}	2.89 ± 1.38^{b}	2.59 ± 0.52^{b}	2.73 ± 0.61^{b}
	S	5°C	1 ± 0^{ab}	0.73 ± 0.11^{ab}	0.82 ± 0.2^{ab}	0.66 ± 0.29^{ab}	0.49 ± 0.17^{a}	0.6 ± 0.32^{ab}	0.34 ± 0.12^{a}	0.5 ± 0.25^{a}
		15°C	1 ± 0^{ab}	0.69 ± 0.28^{ab}	0.64 ± 0.28^{ab}	0.73 ± 0.15^{ab}	0.7 ± 0.24^{ab}	0.64 ± 0.2^{ab}	0.65 ± 0.21^{ab}	1.06 ± 0.34^{ab}
		25°C	1 ± 0^{ab}	2.08 ± 0.28^{bc}	4.71 ± 0.33^{d}	6.27 ± 1.26^{e}	2.39 ± 0.31^{c}	2.3 ± 0.31^{c}	1.52 ± 0.4^{b}	1.03 ± 0.34^{ab}
	CS	5°C	1 ± 0^{b}	0.85 ± 0.12a^{b}	1.12 ± 0.13^{b}	0.67 ± 0.11^{ab}	0.7 ± 0.35^{ab}	0.48 ± 0.18^{a}	0.55 ± 0.36^{ab}	0.35 ± 0.27^{a}
		15°C	1 ± 0^{b}	1.07 ± 0.16^{b}	0.95 ± 0.11^{b}	1.14 ± 0.2^{b}	1.15 ± 0.1^{b}	1.16 ± 0.07^{b}	1.15 ± 0.15^{b}	1.12 ± 0.1^{b}
		25°C	1 ± 0^{b}	3.67 ± 0.35^{d}	6.8 ± 0.56^{e}	3.62 ± 0.44^{d}	3.34 ± 0.2^{d}	3.29 ± 0.16^{d}	1.99 ± 0.08^{c}	1.79 ± 0.24^{bc}
NyHSP70-2	G	5°C	1 ± 0^{a}	0.45 ± 0.08^{a}	0.44 ± 0.09^{a}	0.45 ± 0.23^{a}	0.16 ± 0.07^{a}	0.35 ± 0.44^{a}	0.4 ± 0.49^{a}	0.34 ± 0.24^{a}
		15°C	1 ± 0^{a}	1.73 ± 1.36^{a}	1.36 ± 0.68^{a}	1.3 ± 0.91^{a}	1.37 ± 0.89^{a}	1.33 ± 0.87^{a}	1.34 ± 0.93^{a}	1 ± 0.34^{a}
		25°C	1 ± 0^{a}	13.9 ± 1.85^{c}	50.2 ± 4.32^{ef}	126.33 ± 12.02^{g}	44.73 ± 4.05^{e}	21.79 ± 2.9^{d}	53.24 ± 4.7^{f}	5.52 ± 2.43^{b}
	S	5°C	1 ± 0^{ab}	0.92 ± 0.14^{ab}	1.14 ± 0.24^{b}	0.82 ± 0.31^{ab}	0.7 ± 0.32^{a}	0.54 ± 0.38^{a}	0.59 ± 0.36^{a}	0.67 ± 0.35^{a}
		15°C	1 ± 0^{ab}	1.23 ± 0.18^{b}	1.27 ± 0.19^{b}	1.16 ± 0.12^{b}	1.34 ± 0.32^{b}	1.28 ± 0.34^{b}	1.02 ± 0.06^{ab}	0.63 ± 0.25^{a}
		25°C	1 ± 0^{ab}	7.27 ± 0.67^{c}	21.91 ± 1.67^{e}	28.51 ± 2.04^{f}	10.12 ± 0.79^{d}	11.52 ± 0.88^{d}	18.39 ± 1.83^{e}	1.88 ± 0.5^{b}
	CS	5°C	1 ± 0^{b}	0.84 ± 0.12^{b}	0.6 ± 0.14^{ab}	0.51 ± 0.46^{ab}	0.14 ± 0.09^{a}	0.44 ± 0.38^{ab}	0.17 ± 0.08^{a}	0.32 ± 0.23^{a}
		15°C	1 ± 0^{b}	0.36 ± 0.09^{a}	0.4 ± 0.16^{a}	0.27 ± 0.13^{a}	0.42 ± 0.18^{ab}	0.39 ± 0.15^{a}	0.34 ± 0.26^{a}	0.58 ± 0.16^{ab}
		25°C	1 ± 0^{b}	3.96 ± 0.35^{c}	14.58 ± 1.15^{e}	7.66 ± 0.51^{d}	3.6 ± 0.41^{c}	1.8 ± 0.28^{b}	8.8 ± 0.74^{d}	1.49 ± 0.43^{b}

Mean values ± SD were calculated from triplicate experiments, and letters denote statistically significant differences ($p < 0.05$) determined by two-way ANOVA. G, gametophyte; S, sporophyte; CS, conchosporophyte.
